# Meanings and mechanisms of One Health partnerships: insights from a critical review of literature on cross-government collaborations

**DOI:** 10.1093/heapol/czab134

**Published:** 2021-11-17

**Authors:** Syed Shahid Abbas, Tim Shorten, Jonathan Rushton

**Affiliations:** Institute of Development Studies, University of Sussex, Falmer, Brighton BN1 9RE, UK; Public Health Foundation of India, Plot No. 47, Sector 44, Institutional Area Gurugram 122002, India; Independent Priory Farm, Half Moon Lane, Redgrave, Suffolk IP22 1RX, UK; Institute of Infection, Veterinary and Ecological Sciences, University of Liverpool, Neston, Liverpool CH64 7TE, UK

**Keywords:** Multisector, intersectoral, collaboration, partnerships, integration, One Health, AMR

## Abstract

Complex health policy challenges such as antimicrobial resistance and other emerging infections are driven by activities in multiple sectors. Therefore, addressing these also requires joint efforts from multiple sectors as exemplified in the One Health approach. We undertake a critical review to examine the different ways in which multisector partnerships have been conceptualized across multiple disciplines and thematic areas. We started with a set of six articles from the disciplines of health, nutrition and public administration that reviewed conceptual frameworks within their respective fields. We conducted backward citation tracing using the bibliography of the six articles to identify other articles in the same and related fields that conceptualized multisector partnerships. We identified 58 articles published from 1967 to 2018 from the fields of global health, infectious diseases, management, nutrition and sustainability sciences indicating that multisector partnerships have been a topic of study across different fields for several decades. A thematic analysis of the 58 articles revealed that multisector partnerships assume a variety of forms and have been described in different ways. Partnerships can be categorized by scope, scale, formality and strength. Multisector partnerships emerge in conditions of dynamic uncertainty and sector failure when the information and resources required are beyond the capacities of any individual sector. Such partnerships are inherently political in nature and subsume multiple competing agendas of collaborating actors. Sustaining collaborations over a long period of time will require collaborative approaches like One Health to accommodate competing political perspectives and include flexibility to allow multisector partnerships to respond to changing external dynamics.

**Key messages**

- Multisector collaborations are complex political enterprises, which incorporate within themselves, different visions around, objectives, mandates, function and success.
- Collaborations can take various forms which allow practitioners to choose the form of collaboration that is most relevant to their requirements.
- Most collaborations consist of partners who pursue different individual and institutional agenda and who exercise different amounts of influence. Collaborations should learn how to manage rather than minimize these differences.
- While there are no fixed parameters for success, given the dynamic contexts within which multisector collaborations function, they can result in unintended outcomes. Collaborations that are responsive to changing internal and external dynamics are more likely to thrive.

## Introduction: need for review

### About One Health

The ongoing coronavirus disease-2019 (Covid-19) pandemic has highlighted the importance of and need for multisector action for research, preparedness as well as response to pandemics (Häsler *et al.*, 2020; Ruckert *et al.*, 2020). Consequently, the last 2 years have seen multiple calls for One Health approaches to inform research as well as decision-making at the global level (Amuasi *et al.*, 2020; Department of Health and Social Care, 2021).

The term One Health refers to an approach that seeks to foster interactions among animal, environment and human health sectors, as well as the disciplinary fields associated with them. Initially framed by conservationists in the context of wildlife-origin emerging infectious diseases in 2003, the concept of One Health quickly gained currency, especially in discussion related to pandemic preparedness (Osofsky *et al.*, 2003; IMCAPI, 2010). It was adopted by a tripartite initiative of the Food and Agriculture Organisation (FAO), the World Organisation for Animal Health (OIE) and the World Health Organisation (WHO) as a way of mobilizing joint action, which expanded the ambit of One Health to cover enzootic infections and antimicrobial resistance (AMR) (United Nations, 2008; Food & Agriculture Organization, World Organization for Animal Health, World Health Organization, 2010).

While the term One Health is of recent coinage, the underlying ideas about mutual interdependencies of animal, environment and human health are not. For instance, the One Health approach draws from earlier convergence movements, such as One Medicine and Ecohealth approaches (Zinsstag *et al.*, 2011; Woods *et al.*, 2018). The reasons for repeated calls for One Health in the wake of zoonotic influenza earlier in the millennium, or more recently, AMR and pandemics are not hard to guess, as discussed below.

### Wicked problems and One Health

The problems of zoonotic infections, pandemics and AMR have been referred to as key challenges facing humanity in the 21st century and associated with increased risks to global security, poverty, food security, health and development (Perry and Grace, 2009; Davies, 2013; De Bengy Puyvallée and Kittelsen, 2019; Laborde *et al.*, 2020; Ottersen and Engebretsen, 2020). In addition to their magnitude, one of the peculiar challenges of zoonoses and AMR is that the problem emerges from and affects multiple sectors—such as pharmaceuticals, crop and animal agriculture, food processing, water resources and public health, among others. These sectors operate under different incentive structures and are informed from diverse disciplinary perspectives.

**Figure 1.**
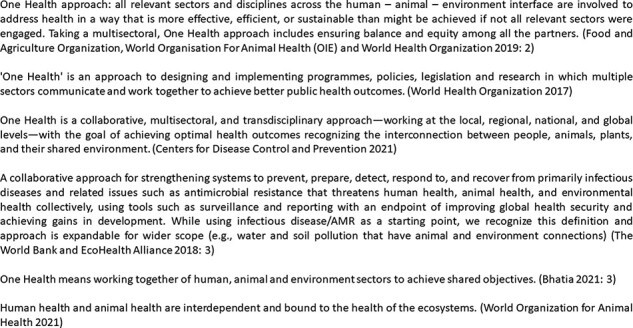
Definitions of One Health advanced by key international agencies

The problem of infectious diseases can therefore be framed from a variety of perspectives with often competing, sometimes incompatible, understandings. As such AMR and zoonoses can be viewed as ‘wicked problems’ whose solutions are difficult to clearly define (Waltner-Toews, 2017; Legido-Quigley *et al.*, 2019). As is the case with other similarly deeply entrenched problems that lend themselves to multiple and incomplete characterizations—such as climate change—many believe combating AMR and zoonoses requires the presence of a ‘transdisciplinary imagination’ that can go beyond singular, incomplete perspectives offered within individual disciplines (Harris *et al.*, 2010). In an institutional context, this has been referred to as ‘sector failure’—when the problem is beyond the capacity or complexity for one sector to handle alone (Bryson *et al.*, 2006, p. 46). Acknowledging the multi-sectoral distribution of AMR, for instance, and its ability to spread rapidly across species and countries, Robinson *et al.* (2016) have termed it as a ‘One Health’ as well as a ‘One World’ issue. Actions to combat a problem that is both multi-sectoral and multi-dimensional in terms of cutting across disciplines require interdisciplinary response—One Health approaches, variously defined in Figure 1, would fit this description. Therefore, not surprisingly, the lead global agencies for global animal, environment and human health identified AMR and zoonoses as ‘Entry Points’ for a One Health approach (Food & Agriculture Organization, World Organization for Animal Health, World Health Organization, 2010), and the UK-funded Fleming Fund programme has adopted One Health as one of its underpinning principles for addressing AMR (UK Department of Health & Social Care, UK Aid, 2018, p. 5).

### Challenges facing One Health

However, establishing multisector thinking underpinned by an interdisciplinary culture is often challenging as it runs counter to the established cultures of specialization and silo-based compartmentalizations prevalent within academic and governmental bureaucracies (Valeix, 2018; Surdez *et al.*, 2021). This is perhaps why more than 17 years after the term ‘One Health’ was coined (Osofsky *et al.*, 2003), its operationalization continues to be a challenge (Lee and Brumme, 2012; Okello *et al.*, 2014) and there continues to be a strong demand for frameworks to operationalize the approach (United Nations, 2008; Centers For Disease Control and Prevention, 2010; The World Bank, EcoHealth Alliance, 2018; Food and Agriculture Organization, World Organisation For Animal Health (OIE), World Health Organization, 2019).

Most One Health guidelines promote formal, institutional collaborations and a singular health-dominated agenda for action. The increased demand for One Health programmes is in contrast with the limited nature of discussion around the attributes, quality or drivers of One Health collaborations. This simplistic understanding of collaborations runs the risk that One Health is seen more as rhetoric than a series of actions leading to practical solutions (de Garine-wichatitsky *et al.*, 2020).

The differences in the expectations from One Health are borne out of the definitions of One Health advanced by major international agencies which can be assumed to be representative of their respective sectoral interests (see Figure 1). For instance, the Centers for Disease Control and Prevention and WHO definitions of One Health emphasize public health achievements as shared outcomes for One Health partners (World Health Organization, 2021; Centers for Disease Control, 2021). The World Bank definition also gives primacy to global health security but allows for expanding the scope to other non-health outcomes for One Health (The World Bank, EcoHealth Alliance, 2018, p. 3). The definitions from the two non-health agencies, the FAO and OIE, go beyond purely health outcomes and adopt a more open-ended note. While the FAO definition cites ‘shared objectives’ instead of health outcomes, the OIE definition merely recognizes the interconnectedness of different sectors without specifying specific outcomes (Bhatia, 2021; World Organization for Animal Health, 2021). These definitions seem to reflect the remit and interests of their respective organizations and varying levels of interest in purely human health outcomes. Not surprisingly, the apparent lack of clarity in One Health vision has led many commentators to highlight the ‘moral dilemma of One Health’; i.e. whose world and whose health does the framing seek to capture (Galaz *et al.*, 2015; MacGregor and Waldman, 2017; van Herten *et al.*, 2019)?

The limited nature of theorization of collaborations is not unique to AMR or One Health alone, but extends to other avenues for intersectoral action involving health sector as well (Hendriks *et al.*, 2014; Chircop *et al.*, 2015; Dubois *et al.*, 2015; Glandon *et al.*, 2019). Given the limited theorization of collaboration within One Health literature, we cast a wide net to conduct a broad-based review across the disciplines of global health and public administration. In this critical review, we aim to identify what are the different conceptual understandings around collaboration and discuss their relevance to discussions around AMR and zoonoses control.

Accordingly, we have structured the paper in three sections. The Methods section describes and justifies the choice of study design, search strategy, inclusion criteria and analysis plan. The following section on Results describes how multisector collaborations have been discussed in literature around the themes of infectious diseases, global health, nutrition, management studies and public administration. The Discussion section leads with an examination of the diversity and categorization of collaborations. This is followed by a discussion of key drivers of multisector collaborations as viewed from a complex systems lens. The last part of Discussion returns to the themes of AMR and One Health and reflects on possible lessons for developing the field of One Health science and practice.

## Methods

### Critical review

We use a critical review design to examine the different ways in which multisector collaborations have been conceptualized across multiple disciplines and thematic areas. While standard systematic reviews require a precisely formulated question which are mostly directed towards establishing treatment effect or causality, they do not lend themselves to critically appraising the assumptions and perspectives informing individual studies (Grant and Booth, 2009; Higgins *et al.*, 2019). We found critical reviews to be better suited for our study since our ambition was to hold up ‘each work against a criterion’ and not to ‘summate conclusions or compare the covered works one to another’ (Cooper and Hedges, 2009, p. 5).

This review focusses on conceptual understandings of collaborations, which could help us draw inferences for AMR and disease control policies. Since we were starting from a point of relatively limited theorization and as we were surveying literature from a broad set of areas adopting a critical review approach allowed us to conduct a broad survey of the extant literature ‘to reveal weaknesses, contradictions, controversies, or inconsistencies’ (Paré *et al.*, 2015, p. 190).

### Search strategy

The review is not meant to be exhaustive; rather it casts a wide net to capture a breadth of understandings around cross-government collaborations and discuss their relevance to disease control in Low and Middle Income countries (LMICs). We use a selective search strategy with the intention of capturing more visible papers across several disciplinary fields and supplemented these with backward citation tracing.

Citation searching can provide a supplementary or an alternative search strategy to the standard keyword-based search techniques. These are of two types. Backward citation tracing uses the bibliography from a source study to identify additional studies. This is opposed to a ‘forward citation tracing’ which traces articles citing the source study (Briscoe *et al.*, 2020). Citation tracing helps consolidate searches from multiple studies and is therefore likely to yield increased and better-quality search results when compared to structured keyword-based searches (Kuper *et al.*, 2006; Hinde and Spackman, 2015; Hirt *et al.*, 2020).

Citation searches can be especially useful when the core concepts are difficult to define or have been variously defined, as we posit has been the case with multisectoral One Health collaborations (Briscoe *et al.*, 2020). These can be a powerful tool for explorative analysis for small bodies of literature (Akin, 1998). In addition, starting citation searches from an index review tends to yield a limited and relevant body of literature (Kuper *et al.*, 2006).

Accordingly, we started with a small set of papers from a broad set of fields that included global health (infectious diseases, social determinants and nutrition) and public administration disciplines (see Table 1). These were purposively selected articles that reviewed a body of conceptual literature within their respective field as well as presented their own depictions of multisector collaborations. These represented the starting points of our inquiry which would allow us to broaden the scope of our review and overcome the limitations of a backward citation tracing. We then identified the papers cited in the reviews (backward citation tracing) to locate additional papers within their respective fields. These searches were conducted between June 2019 and January 2020.

**Table 1.** Review articles selected for backward citation tracing

Review paper	Domain
(Emerson, 2018)	Health and Public Administration
(Glandon *et al.*, 2019)	Health (Health systems research)
(Harris and Drimie, 2012)	Nutrition
(Kuruvilla *et al.*, 2018)	Health (Maternal and Child Health)
(Mahlangu *et al.*, 2019)	Health (HIV/AIDS and Public Health)
(Thomson *et al.*, 2007)	Public administration

### Inclusion criteria

While recognizing that multisector collaborations might assume different forms, including *ad hoc* and informal ones, for the purpose of this review, we restricted ourselves to literature around formal, institutional modes of collaborations. This was to reflect the priorities of current One Health guidelines from international technical agencies which promote institutionalized partnership mechanisms (World Health Organization, 2015).

The second inclusion criterion related to conceptual contributions. Original research studies, monographs, reviews, technical reports as well as conceptual papers were all included in the review as long as they contributed to conceptualization of dynamics of multisector collaborations—such as processes, drivers and outcomes. Empirical studies that did not generalize their findings beyond their immediate context were not included.

Since a substantial number of references were published books or programme documents, we excluded those publications whose full text could not be accessed.

### Thematic analysis

Thematic analysis offers a flexible approach to organizing and describing qualitative data. In addition, it also allows the researcher to identify patterns and perform interpretations (Braun and Clarke, 2006; Nowell *et al.*, 2017). We used thematic analysis to first organize the data based upon the key domains or topics addressed within them. This allowed us to describe the broad trends on how multisector collaborations have been addressed within the field and identify the key concerns that these debates set out to address.

We describe key debates around multisector collaborations with the fields of infectious diseases, global health (including the topics of women’s and children health, and social determinants of health), nutrition, management studies and public administration. This list of fields does not exactly tally with the domains of the initial list of six articles (Table 1) since we had to accommodate a number of papers and the richness of debates in different often overlapping fields.

The next focus of our inquiry was on examining the diversity of collaborations covered in the literature. We found different ways in which collaborations have been described and chose to focus on two categorizations (formality and strength). Finally, we wanted to understand the functioning of the collaborative process itself. This included an examination of the structure of the collaborative systems—and using a temporal lens—to assess how collaborates originate, operate and sustain. We used the shortlisted publications to structure our inquiries and supplemented these with additional literature as we proceeded to develop our explanations.

## Results

### Literature landscape

**Figure 2.**
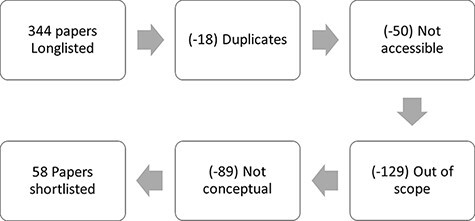
Identifying publications for review

As described in Figure 2, our search generated an initial long list of 344 publications. From these we excluded duplicate references (18) and inaccessible publications (50). The relatively large number of inaccessible publications can be explained by a substantial number of books (30), technical reports (12) and conference presentations (5), included in this figure, which were not accessible electronically.

A total of 129 papers were found to be out of scope of our review. These included methodological, contextual or reference literature and did not contribute to conceptual discussions on collaborations. Finally, 89 publications were excluded which were mostly empirical studies or made general observations on collaborations.

While it is difficult to strictly classify the disciplinary fields for these publications, based upon a loosely applied categorization, most of the shortlisted research publications appear to be from public administration and related field of management studies. Most of the publications were published recently; this includes more than half the papers which were published over the last decade (Table 2).

**Table 2.** Distribution of shortlisted papers, by domain and decade

Domain	Publications	Decade	Publications
Public administration	26 (45%)	1960–1969	1 (2%)
Health	13 (22%)	1990–1999	8 (14%)
Management	12 (21%)	2000–2009	19 (33%)
Sustainability sciences	4 (7%)	2010–2019	30 (52%)
Nutrition	3 (5%)	2020–2021	0 (0%)
Total	58 (100%)	Total	58 (100%)

### Depictions of multisector collaborations

The idea of multisector collaborations has been variously referred to by related terms such as integration, partnerships and collaboration as well as multi-sector and inter-sector. All these terms refer to related but distinct concepts of working together. In some cases, collaborations are considered akin to partnerships (World Health Organization South East Asian Regional Office, World Health Organization Western Pacific Regional Office, Food & Agriculture Organization *et al.*, 2008) and integration as a more ‘joined-up’ way of working (Davies, 2009; Exworthy and Hunter, 2011). In other contexts, multisectorality refers to a more prescriptive form of alignment of different agendas towards a common goal (Shankardass *et al.*, 2015). Even what is referred to as sectors in ‘multisectoral’ varies a lot. As in the case of One Health or nutrition, sectors could refer simultaneously to economic spheres of activity or to different corresponding disciplines (Parkes *et al.*, 2005; The World Bank, 2013; Okello *et al.*, 2014). In other contexts, sectors could refer to the ownership of the institutions—such as state, non-governmental organizations and citizens, as in the case of collaborative governance literature (Tennyson and Wilde, 2000; Emerson *et al.*, 2012). Finally, while we feel there are differences between multisector and intersector approaches (see Figure 4), in much of programmatic literature they have been used interchangeably (World Health Organization, 2018).

**Figure 3.**
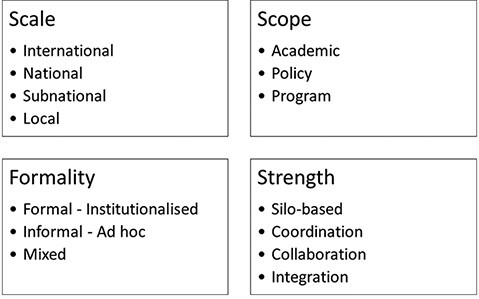
Different ways of characterizing multisector collaborations

**Figure 4.**
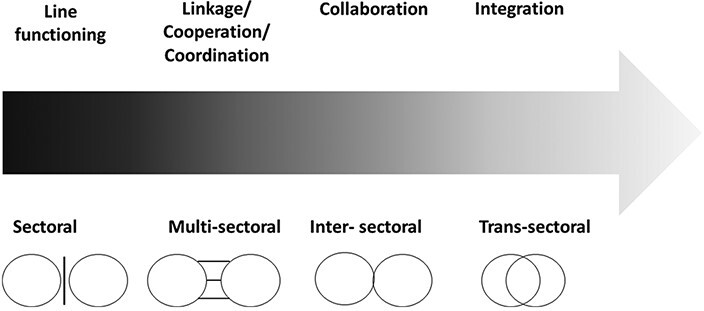
Types of collaborations, by strength; adapted from Harris and Drimie (2012, p. 2)

Therefore, given the lack of established typologies around multisector collaboration, we have considered all the above labels as referring to similar ideas for the purpose of this review. This section explains the different ways in which the multisector collaborations have been addressed in literature relating to infectious diseases, nutrition, global health, management studies and public administration.

#### Infectious diseases

Given the ubiquitous nature of infectious diseases, the tendency of microbes to cross geographic and species boundaries was well understood from the beginnings of modern medicine (Schultz, 2008; Woods, 2017). It is therefore not surprising that discussions around multisectorality have taken place widely, and for long, across different pathogens. As described below, this includes discussions around integrated disease surveillance, food-borne diseases, Neglected Tropical Diseases (NTDs), Zoonoses, Emerging Infectious diseases (EIDs), Antimicrobial resistance (AMR) as well as Human Immunodeficiency Virus (HIV)/Acquired Immune Deficiency Syndrome (AIDS); at a historical level, the successful eradication of zoonotic diseases such as brucellosis from cattle herds in North America and Europe and the control of tuberculosis in cattle in the same regions. These were multisectoral efforts brought about through crisis or recognition by the private sector that coordination was needed (Olmstead and Rhode, 2015).

The early 2000s witnessed a worldwide push for ‘integrated’ disease surveillance efforts that brought together activities across different diseases, and also, across health and non-health sectors (WHO *et al.*, 2003; Calain, 2007). This was a time when the next pandemic was widely anticipated, and global health agencies had started recognizing the limitations of recognizing the limitations of the pathogen-based funding models which were then prevalent (Shiffman 2006). While the surveillance programmes were very much health centric, the literature on infectious diseases and integration recognized the role of non-health factors in disease causation and the importance of addressing these (Atun *et al.*, 2010; Shigayeva *et al.*, 2010). Similarly, the notion of working with non-health sectors to address NTDs was the motivation for a framework conceptualizing a gradient of progressively stronger collaborations, later also reflected upon by Harris and Drimie (2012) in the context of nutrition (Grépin and Reich, 2008).

The increasing awareness of social origins of HIV/AIDS prompted early thinking on engagement with non-health sectors (Marks, 2002; O’Neil *et al.*, 2004). While many innovative approaches were developed for identifying and engaging with non-health sectors, the objectives of these programmes were largely restricted to outcomes focussed upon single pathogens and within health sector alone (Kar, 2014; Mahlangu *et al.*, 2018). The unique visibility of HIV/ADS programmes, however, resulted in distortion of local health systems priorities in many affected countries (Biesma *et al.*, 2009; Sherr *et al.*, 2012), which was a major factor in call for integrative health systems wide support and integration with local health priorities (Frenk, 2009; Shakarishvili *et al.*, 2009; Gyapong *et al.*, 2010).

Alongside the above discussions, other convergence movements gathered momentum around infectious diseases. Prominent among these included the Ecohealth movement. It drew connection between human health with different components of ecosystems, including biological and physical environment (Schwabe, 1984; Parkes *et al.*, 2005; Waltner-Toews and Kay, 2005; Charron, 2012). This was closely related to the newly launched One Health approach (Zinsstag *et al.*, 2011). The term ‘One Health’ itself was coined in 2003 and was largely framed as a response to potential threats from wildlife-origin diseases emanating from the risk hotspots of the global south (Leach *et al.*, 2010; Cassidy, 2018).

As pointed out earlier in the paper, much of the One Health literature is still concerned with establishing the need for collaboration (The World Bank, 2010; 2012; Gebreyes *et al.*, 2014), or in advancing standardized frameworks for implementation (World Health Organization South East Asian Regional Office, World Health Organization Western Pacific Regional Office, Food & Agriculture Organization *et al.*, 2008; The World Bank, EcoHealth Alliance, 2018; Food and Agriculture Organization, World Organisation For Animal Health (OIE), World Health Organization, 2019). While there have been some attempts at theorizing One Health such as by the use of socio-ecological models (Wilcox and Kueffer, 2008; Zinsstag *et al.*, 2011; Rüegg *et al.*, 2018a), thinking around the politics and mechanics of One Health continue to lag behind (Lee and Brumme, 2012; Hinchliffe, 2015; Ssennyonjo *et al.*, 2021). Human health priorities tend to dominate most discussions around One Health, and most benefits are still assessed from human health perspective, even in ostensibly One Health analysis (Zinsstag *et al.*, 2007; Fitzpatrick *et al.*, 2016).

#### Global health

Some of the strongest calls for multisector engagement from the health sector have come from work done on social inequalities, specifically around the social determinants of health (Commission on Social determinants of Health, 2008; Public Health Agency of Canada, Commission on Social Determinants of Health, Regional Network for Equity in Health in East and Southern Africa, 2008). These calls for intersectoral action were reframed by Finland during its European Union presidency as a call to include ‘Health in All Policies’ in 2006. This eventually led to the Helsinki statement during the 8th Global Conference on Health Promotion in 2013 (Puska, 2007; Greszczuk, 2019).

However, despite the visibility of the approach, Health in All Policies has frequently faced implementation challenges. An examination of the governance challenges facing the approach recommended going beyond rigid bureaucracies and adopting a collaborative style of governance (Mcqueen *et al.*, 2012). A recently published assessment identified several challenges to the Health in All Policies approach, such as mismatched incentives and competing priorities of collaborating partners within which it was easy to get the focus on health equity diluted (Greszczuk, 2019). Shankardass *et al.* (2015), (2018) proposed using systems theory and realist evaluations to characterize the complexity of multi-agency collaborations and capturing the tensions noted above.

The explicit focus on partnerships and convergence within the SDGs, along with the insights from earlier debates described above, have helped inspire a range of new scholarly efforts to think about intersectoral action within the context of child health (Blomstedt *et al.*, 2018; Kuruvilla *et al.*, 2018) and health systems research domains (Dubois *et al.*, 2015; Rasanathan *et al.*, 2015; 2017; Bennett *et al.*, 2018; Graham *et al.*, 2018). Arguably, the key advance in some of these efforts has been increased reflection on the theory (Glandon *et al.*, 2019) as well as incorporation of insights from non-health disciplines including public administration (Rasanathan *et al.*, 2017; Emerson, 2018).

For instance, Glandon *et al.* (2018), (2019) aimed to develop a research agenda around multisector collaborations. While their initial study on research priorities found a long-held and widespread interest in studying multisector collaborations from a wide range of disciplines and geographies, they found that fundamental questions about initiation, composition, functioning and governance of multisector collaborations remained unclear. Their follow-up study identified the need for development of more theory-driven and outcome-focussed research, and for critical examination of the motivations of collaborating partners within multisector collaborations (Glandon *et al.*, 2018; 2019). Kuruvilla *et al.* (2018) conducted a theory-driven analysis of 12 country case studies of multisector collaborations to identify the different forms such collaborations could take and develop a model outlining the enabling factors that allowed multisector collaborations to respond to changing external context and internal dynamics.

#### Nutrition

As a sector that has to interact closely with agriculture and health, nutrition has had a long history of promoting and participating in multisector platforms. Indeed, some of the earliest examples of large-scale integrated programmes came from nutrition, such as the Integrated Child Development Services programme in India (Rajivan, 2006). Multisector collaborations had already had a long history by the 80s, when nutrition scholars were debating the possible ‘demise’ of and relative merits of ‘multisector planning’ in nutrition (Berg, 1987).

Perhaps because of their long-standing practical exposure to collaboration, many nutrition scientists advocate nuanced perspectives on multisectorality. While recognizing the arguments of efficiency and effectiveness underpinning justifications for multisectoral collaborations in nutrition, they point to strong cultural challenges facing such initiatives. Harris and Drimie (2012, p. 1) highlight the presence of many nutrition-specific interventions that tend to be ‘owned’ by health department. This has resulted in many multisector efforts to be conducted piecemeal within individual sectors (Garrett and Natalicchio, 2011, p. 8). However, these scholars also point to the presence of small, under-analysed examples of multisector efforts whose lessons for mechanics of multisector collaborations need to be better understood.

#### Management studies

Much work around collaborations within private sector management has occurred around team management (Gratton and Erickson, 2007). However, there is a rich vein of literature unpacking the practical elements of collaborations by social management and organizational management scholars.

The efficiency motivation behind collaborations is frequently cited to make a case for donor alignment or for multiple institutions to work together as in the case of PPPs or collaborative management (Kalegaonkar and Brown, 2000; Tennyson, 2003).

Another discussion strand within the management discipline has been around recognizing the differences in powers and interests within collaborating partnerships and how to approach building agreements using consensus-driven and power-aware approaches (Innes, 1999; Brouwer *et al.*, 2013).

Recognizing the challenges involved in creating and sustaining collaborations across organizations, some management scholars describe these as ‘collaborative inertia’. On the other hand, if collaborations are nurtured by addressing issues of conflicting aims, power dynamics and fostering trust, for example it is possible to achieve ‘collaborative advantage’ or a state of synergy that organizations can aspire towards when working together (Huxham, 2003; Huxham and Vangen, 2004; Lank, 2006). They also recognize the transformative potential of such collaborations and offer practical ways for organizations to instil a culture of collaboration (Brouwer *et al.*, 2016).

#### Public administration

As a discipline that is tasked with the study of ‘organization and management practices in collective or public settings’, public administration scholars have unsurprisingly been studying multisector collaborations deeply and more widely than other fields (Frederickson *et al.*, 2012, p. 1)—an early attempt to develop a theory for collaborations being in 1991 (Gray and Wood, 1991; Wood and Gray, 1991).

Much of the work in the field in the last few decades has led to the recognition of the limitations of the state as an actor. This has accompanied an increasing recognition of the importance of engaging non-state actors, such as civil society organizations, private sector and academia in public policy planning and implementation (Peters and Pierre, 1998; Bourgon, 2011).

This has resulted in new thinking around collaborative public management (McGuire, 2006) and intergovernmental or interagency collaboration (Hudson *et al.*, 1999; Agranoff, 2007; 2013) that seek to encourage local governments to engage with governmental and non-governmental stakeholders (Crosby and Bryson, 2005; Bryson *et al.*, 2006; Page *et al.*, 2015).

As discussed in the section on systems thinking below, many of these scholars have theorized multisector collaborations as complex systems, which has resulted in new insights on how to develop a typology, drivers and elements of such collaborations (Crosby and Bryson, 2005; Bryson *et al.*, 2006; Ansell and Gash, 2007; Emerson *et al.*, 2012; Emerson and Gerlak, 2014; Emerson and Nabatchi, 2015; Page *et al.*, 2015).

## Discussion

We have structured the Discussion section in three parts. The first part discusses possible typologies of multisector collaborations. The second part examines possible drivers of multisector collaborations informed by a complex system understanding of collaborations. The last part draws upon the collaboration literature in other fields to draw lessons for the One Health movement.

### Collaborations come in different shapes and sizes

Based on an examination of the literature around collaborations cited above, it is clear that the concept has been continued to be discussed in different ways across multiple disciplines and applications. To cite an example, even as Berg and Field were debating the strengths and limitations of multisectoral approaches in nutrition in late eighties (Berg, 1987; Field, 1987), around the same time Gray and Wood were putting together a special journal issue aimed at developing a comprehensive theory of collaborations (Gray and Wood, 1991; Wood and Gray, 1991).

Multisector collaborations have been characterized in many ways in the reviewed literature. The different descriptors can be broadly grouped under four different ways, depicted in Figure 3. Two relatively straightforward ways to categorize collaborations are by describing the scale and scope of their activities. Collaborations can be termed as international, national or subnational depending upon the sphere of their activities, for instance. Similarly, collaborations can also be said to focus on programme, policy or research depending upon their scope of functioning.

The above two ways of categorizing collaborations have the advantage of describing the nature of activities of collaborative enterprises. However, these classifications do not shed any insights on the inner processes of the collaborations themselves, such as the factors precipitating and sustaining collaborations. The two other ways of classifying collaborations—based on their formality and strength can be more informative in this regard, as explained below.

#### Degree of formality

Even though most analyses of collaborations recognize how collaborations are underpinned by inter-personal dynamics (Agranoff, 2006; Bryson *et al.*, 2006; McGuire, 2006; Ansell and Gash, 2007; Harris and Drimie, 2012) and are shaped by institutional and social norms (Emerson, 2018), the importance of informal collaborations remains a topic of ongoing debate. While some argue about the importance of informal relationships (Bryson *et al.*, 2006; Chenard, 2007), and recognize that formal and informal components are both parts of the ‘collaborative black box’ (Thomson and Perry, 2006), others label informality as ‘Organizational individualism’ that are not appropriate for institutional collaborations (Hudson *et al.*, 1999).

Some management scholars provide a more sophisticated view of formality. For instance, Tennyson (2004, p. 14) recognizes that there can be degrees of informality within collaborations and that there would be relative pros and cons of different levels of formality. An examination of One Health collaborations in India found that formal and informal collaborations often go hand in hand. Formal collaborations are often underpinned by the leadership and agency from participating members, while informal collaborations seldom persist without some level of tacit approvals from the bureaucracy (Abbas, 2018).

#### Strength of integration

Harris and Drimie (2012) propose a scheme of categorizing collaborations by their strength of integration that resonates with several other conceptualizations of multisector collaborations as well (Kalegaonkar and Brown, 2000; Boon *et al.*, 2004; Grépin and Reich, 2008; Shigayeva *et al.*, 2010).

As described in Figure 4, which is adapted from Harris and Drimie (2012, p. 2), all multisector engagements can be placed on a gradient of progressively more integrative partnerships. This gradient starts from the left where sectors continue to work in individual silos. This leads on to the stage of cooperation or coordination which might involve exchange of information, and possibly joint planning with each sector conducting its own activities in the field independently and maintaining strong sectoral boundaries. The next stage of collaboration moves beyond unstructured cooperation or coordination to conducting joint operations in the field, and even exchange of personnel and use of shared resources. The last stage is termed as integration which is characterized by the physical merging of resources and a blurring of the sectoral boundaries both in terms of resources, as well as sectoral remits.

It is important to note here that the categorizations discussed above provide a vocabulary to help us characterize the diversity in different collaborations and do not imply a value judgement on the desirability or preference for a certain kind of partnerships. It is possible that traditional sectoral boundaries might work better in some contexts than in others, depending upon local institutional and political cultures. Instead, we intend to use these categorizations to make the case against adopting a one-size-fits-all approach and for highlighting the need for designing collaborations that are more relevant to their immediate contexts.

### Systems view of multisector collaborations

Multisector collaborations have been frequently described as complex systems consisting of multiple independent parts (Thomson and Perry, 2006; Ansell and Gash, 2007; Emerson *et al.*, 2012; Shankardass *et al.*, 2015; Rüegg *et al.*, 2018b; Mahlangu *et al.*, 2019). This depiction allows us to impose a sequential flow on different collaborative processes—described in one instance as: initiation, consolidation and reflection (Brouwer *et al.*, 2016), or initiation, coordination and durability (De Leeuw, 2017). Others use a systems approach to describe the drivers of collaborations from the lens of inputs, process and outcomes (Thomson *et al.*, 2008; Emerson *et al.*, 2012).

Once we can look past the different sets of terminologies used, there is considerable thematic overlap among the frameworks on the antecedent, starting conditions or system contexts that give rise to collaborations. It is generally accepted that collaborations are initiated or precipitated in crisis situations (Tennyson, 2003) to take advantage of power-resource-knowledge asymmetries among the partners in such a way that the collaboration becomes greater than its sum. Initial trust levels including any prehistory of cooperation or conflict are all useful predictors of collaboration (Ansell and Gash, 2007; Thomson *et al.*, 2007; Emerson *et al.*, 2012).

Emerson *et al.* (2012) provide possibly the most granular understanding of the collaborative process itself. They distinguish between drivers and system contexts as those responsible for initiating collaborations and describe collaborative dynamics as consisting of principle engagement, shared motivation and capacity for joint action. They also link collaborations to adaptive capacities and provide a typology of outputs and outcomes expected from successful collaborations. In this conceptualization the outputs are focussed on the immediate products from the collaboration focussed on the task at hand. On the other hand, the collaborative outcomes and impact will influence changes at a systemic level, and ultimately lead to some form of adaptation (Emerson and Gerlak, 2014; Emerson and Nabatchi, 2015).

This broadly echoes Thomson and Perry (2006, p. 21) assertion, citing other scholars, that outcomes from collaborative undertakings not only include the achievement of immediate goals, but also a transformation of instrumental transactions among organizations into socially embedded relationships; the creation of ‘new value partnerships’ and ‘self-governing collective action’ (Ostrom, ; Ring and Van De Ven, 1994; Sagawa *et al.*, 1999). These insights have implications on what we understand as successful collaborations, and an acknowledgment of the transformative potential of multisector collaborations, as explained below in the section on sustaining collaborations.

#### Principles of multisector collaborations

Many frameworks in the reviewed literature adopt a systems approach for describing multisector collaborations. This means that collaborative process is often categorized into development and operational phase, with different sets of factors driving both (Chen, 2010; Tonelli *et al.*, 2018). As mentioned below, there is limited discussion in the literature regarding identifying the expected outcomes of collaborative process itself, which means there are still open-ended questions regarding the long-term ‘success’ of collaborations (Bryson *et al.*, 2006; McGuire, 2006; Ansell and Gash, 2007; Emerson *et al.*, 2012).

Following the conceptual frameworks mentioned in the key publications, we have described below some of the factors of multisector collaborations, grouped into three phases of collaborative process.

#### Development of collaborations

The conventional wisdom around formal collaborations mentions having a common goal, well-developed prior relationships and ring-fenced objectives. However, most conceptual analysis of multisector collaborations discusses instead the principles of mutual interdependency and complementarity among collaborating partners.

Tennyson *et al.* (2008, p. 16) describes the need for common vision as an ‘enduring myth’ around collaborations. Instead, many researchers of collaboration posit that even when partners come together to collaborate, they do so only to pursue their individual organizational aims. This usually happens when they are compelled to come together by extraordinary circumstances, such as sector failure (Bryson *et al.*, 2006) and uncertainty (Emerson *et al.*, 2012).

Thus, collaborations tend to work best when the strengths and weaknesses of collaborating agencies complement each other and created mutual interdependencies (Bryson *et al.*, 2006; Ansell and Gash, 2007). This provides strong institutional incentives for collaborations to sustain even in the absence of individual champions.

#### Performance of collaborations

Once a collaboration comes into existence, the next challenge is to ensure its performance. In the absence of prior relationships, some argue, trust and a sense of achievement can be built by a series of small wins and informal deals that do not require much trust (Bryson *et al.*, 2006; Ansell and Gash, 2007). The collaborative process tends to be iterative (Ansell and Gash, 2007) involving discovery, definition, deliberation and determination (Emerson *et al.*, 2012).

Observations from cases of collaboration often highlight the role of charismatic leadership as a facilitator (Hudson *et al.*, 1999; Ivery, 2010; Brouwer *et al.*, 2016). Yet, it is possible to break this quality down. Indeed, according to some, it is essential to move beyond the notion of individual charisma if the collaboration is to be sustainable (Tennyson, 2003). Leadership of collaborations require skills in boundary spanning (Quick and Feldman, 2014; Pelletier *et al.*, 2018) and knowledge brokering (Bay *et al.*, 2016; Hitziger *et al.*, 2018) to facilitate communication and compromise across different knowledge systems Also importantly, leaders need to recognize power hierarchies that appear in any group so that they can actively address these to ensure multisectoral action (Purdy, 2012; Brouwer *et al.*, 2013). Ran and Qi (2018, p. 836) are more direct when it comes to power hierarchies and state that *‘*instead of focusing on the attempt to balance power and share power in collaboration, it will be more fruitful to design and implement collaborative arrangements based on the dynamic contingencies’.

#### Sustaining collaborations and building resilience

Unlike the descriptions on antecedents and processes of collaborations, discussion is more sparse on the outcomes expected from collaborative processes. This is possibly a reflection of the instrumental nature of most collaborations where the focus is on immediate deliverables rather than longer-term changes. Most sector-specific literature focus on immediate outcomes such as nutritional status (Levinson and Balarajan, 2013) or on surveillance outputs (Ogyu *et al.*, 2020). It can be argued that while these ring-fenced outcomes might allow the goals to be clearly visualized, these run the risk of artificially smoothening the diversity of interests and agendas at play in a multisector collaboration. Huxham and Hibbert (2008) point out that over the course of their activity, collaborations might result in unanticipated results and outcomes flowing from changed external dynamics or as a result of negotiation within the collaboration itself. In such a situation, those collaborations are likely to thrive that have the space to innovate and seize opportunities (Thomson and Perry, 2006; Tennyson *et al.*, 2008).

On the other hand, there is also evidence to show that both congruence as well as divergence in goals might contribute to success of the collaborative enterprise depending upon the local context (Vangen and Huxham, 2012).

Several scholars argue for the need to institute at least limited autonomy within collaborating partners to allow them to redefine their objectives in light of changing circumstances as well as to allow them to navigate their organization aims (which might have limited overlaps with other partners) (Thomson *et al.*, 2008; Emerson and Gerlak, 2014; Brouwer *et al.*, 2016). If a collaboration does not have the space to go beyond its foundational terms of reference, the collaboration itself is likely to die out when the terms become out of sync with external environment (Ramalingam *et al.*, 2008).

**Figure 5.**
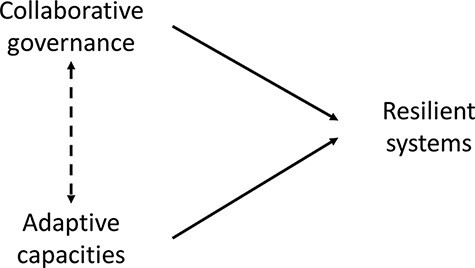
Collaborative systems are resilient systems

This body of work links collaborative governance with adaptive governance (Dietz *et al.*, 2003; Emerson and Gerlak, 2014). As depicted in Figure 5, increased adaptive capacity brought about as a result of multisector collaborations, in turn, is expected to increase the responsiveness and resilience within SES systems (Duit *et al.*, 2010).

On the other hand, adaptive capacities have been associated directly to increasing resilience (within SES). Investing in genuine multisector collaborations and allowing them room to innovate is likely to yield into increasing the responsiveness and resilience of their constituent systems (Dietz *et al.*, 2003; Folke *et al.*, 2005; Duit *et al.*, 2010; Quick and Feldman, 2014).

### Implications for the field of One Health

#### Lessons for One Health science

Having examined the literature around collaborations from other fields, we now return to the domain of One Health. In this section we identify insights from the reviewed literature to examine how we can think about and practice One Health. The complex-systems understanding of multisector collaborations in other fields has broad resonance within One Health as well.

The field of ecosystems approaches to health—which have overlaps with One Health community (Zinsstag, 2012)—has a longstanding tradition of using a socio-ecological lens (Waltner-Toews and Wall, 1997; Charron, 2012) which has informed foundational One Health concepts (Zinsstag *et al.*, 2011; Rüegg *et al.*, 2018a; Rüegg *et al.*, 2018b). However, the enthusiasm for adopting a systems framing has also been tempered by other warnings that these concepts needed to be better internalized across disciplines (Antoine-Moussiaux, 2019; Wilcox *et al.*, 2019; Assmuth *et al.*, 2020). Others have also pointed out the ambiguities in the framing of One Health research and how a lack of recognition of distal causal factors—such as ‘environmental and social injustices driving biodiversity overexploitation and extinction’—will prevent One Health from fulfilling its mandate (de Garine-wichatitsky *et al.*, 2020).

To be sure, recognizing the links between social and ecological systems is merely a starting point in the theorization of One Health. As other examples have shown, more attention to the structural drivers of infections can paint a much richer and more informed picture of how infections occur and are understood by different actors (Wood *et al.*, 2012; Bardosh, 2016; Dzingirai *et al.*, 2017; Wilcox *et al.*, 2019; Thornber *et al.*, 2020). As demonstrated in these examples, in order to design One Health collaborations that better reflect local realities, we will need to have a deep understanding of how different actors perceive diseases and the varying levels of agency exercised by them in order to change their practices.

#### Lessons for the practice of One Health

As the Covid-19 pandemic has demonstrated, decision-making around infectious diseases is often conducted in an environment characterized by uncertainty, dynamism and multi-scale interactions where multiple framings of the disease coexist and compete for attention (Macgregor *et al.*, 2019; Leach *et al.*, 2020). Given the scale and rapidity with which Covid-19 overwhelmed global governance arrangements, this has resulted in increased calls for realignment and coordinated responses—many of which cite One Health as an important tool for responding to the current and future pandemics (Cable *et al.*, 2021; Lal *et al.*, 2021; The Lancet Microbe, 2021).

The current instruments for assessing capacities to respond to disease events such as the International Health Regulations checklist and Performance of Veterinary Services pathways do refer to intersectoral collaborations. While these overlaps have been further enhanced in recent years using the One Health approach (De La Rocque and Formenty, 2014), the ongoing pandemic has led to calls for revisions in the global governance frameworks, and greater coordination among them (Blinken and Becerra, 2021; Lal *et al.*, 2021). In addition, multilateral agencies have developed specific One Health centric guidance to help countries develop their own One Health collaborations (World Health Organization South East Asian Regional Office, World Health Organization Western Pacific Regional Office, Food & Agriculture Organization *et al.*, 2008; The World Bank, EcoHealth Alliance, 2018; Food and Agriculture Organization, World Organisation For Animal Health (OIE), World Health Organization, 2019; Bhatia, 2021).

However, the current One Health guidance is exclusively focussed on formal, institutional mechanisms for initiating multisectoral activities, as illustrated by the example of the AMR Coordination Committee (Seale *et al.*, 2017; Department of Health and Social Care, 2020). Other ways of encouraging interaction and dialogue across bureaucratic silos remain absent in the official literature. Another limitation of the standardized approaches to advocating One Health at the national level is that these fail to take the local context into account. While the newer guidance such as the Tripartite Zoonoses Guide does acknowledge the need for the country programme managers to adapt the approach to their local contexts, there is limited guidance on how to do so (Food and Agriculture Organization, World Organisation For Animal Health (OIE), World Health Organization, 2019).

Given the complex political economy of AMR and zoonoses, disease risks are likely to be understood and responded differently by each of the many different actors affected by them (Leach *et al.*, 2010; Waltner-Toews, 2017; Spruijt and Petersen, 2020). Standardized and formal approaches of One Health governance that fail to recognize these differences in perspectives and operate within bureaucratic hierarchies that do not take into account power imbalances between the partners run the risk of developing blind spots in their management of disease risks. Therefore, the resultant One Health collaborations are not likely to sustain over a long time.

Country-based studies that have examined bureaucratic politics, economic incentives and structural factors governing disease risks have highlighted the importance of these factors in shaping local and national response to disease risks (Hinchliffe *et al.*, 2021; Asaaga *et al.*, 2021; Surdez *et al.*, 2021). Therefore, in order for One Health collaborations to be relevant to local needs, their design and operation should adapt to the role of local politics and informal relationships. In short, for One Health collaborations to succeed, they need to be designed to be locally relevant, be aware of the diversity of interests and influence exercised by the actors involved and allow space for deliberation and adaptation to accommodate changing circumstances and partner needs.

## Conclusions

This review demonstrates that, in addition to the themes of AMR and infectious diseases, multisector collaborations have been proposed and enacted as a policy response to a range of policy challenges that emerge from and impact multiple sectors. These multisector collaborations differ in formats and some will suit a specific context better than others. In their nature, multisector collaborations are complex political enterprises, that incorporate different visions around objectives, mandates, function, and success. Collaborating partners are likely to approach the partnership through their own aims and agenda which might compete or even conflict with the collaborative interests. Therefore, for collaborations to sustain, there will need to be pragmatism and some room for these to manoeuvre and adapt in order to accommodate partner needs or changing circumstances. Such adaptation is more likely to take place when boundaries of the collaboration remain relatively fluid.

Building upon observations from our review, we find several lessons for One Health movement. We suggest that One Health discourse needs to move beyond a one-size-fits-all approach and embrace multiple forms of multisector partnerships that are open to adaptation. In addition, One Health literature should recognise and respond to the politics involved in the development and conduct of multisector collaborations. This includes the diversity of framings within One Health, wherein different partners come to the collaboration with different expectations and agenda. The reference to politics also includes a reference to the different levels of influence enjoyed by the partners to effect change as well as the power dynamics operating among themselves.

A successful collaboration needs to be smart about working with and accommodating this politics instead of ignoring it. We recommend researchers to examine the role of politics in influencing multisector collaborations and that guidance around One Health partnerships from technical agencies being explicit about the political agenda they work in. Rather than standardized approaches, we suggest that the international guidelines on One Health should develop capacities and empower national programme managers to be flexible in choosing a form of One Health that best suits their requirements, and which can accommodate the agenda of different collaborating actors within their respective political landscapes. If the One Health movement can allow and encourage such an adaptive and responsive approach, we suggest, it is more likely that we will see sustainable forms of One Health collaborations to emerge.

## Supplementary Material

czab134_Supp

## Data Availability

The details of reviewed articles in this paper are available as online supplementary material.
